# Recent advances in understanding and treating vasculitis

**DOI:** 10.12688/f1000research.8403.1

**Published:** 2016-06-20

**Authors:** Matthew J. Koster, Kenneth J. Warrington

**Affiliations:** 1 Department of Internal Medicine, Division of Rheumatology, Mayo Clinic College of Medicine, Rochester, MN, 55905, USA

**Keywords:** systemic vasculitides, ANCA-associated vasculitides, vasculitis, B-cell

## Abstract

Anti-neutrophil cytoplasmic antibody (ANCA)-associated vasculitides (AAVs) are near universally fatal conditions if untreated. Although effective therapeutic options are available for these diseases, treatment regimens are associated with both short- and long-term adverse effects. The recent identification of effective B-cell-targeted therapy with an anti-CD20 monoclonal antibody has transformed the treatment landscape of AAV. Questions, nevertheless, remain regarding the appropriate timing, dose, frequency, duration, and long-term effects of treatment. The aim of this article is to provide an overview of the current information, recent advances, ongoing clinical trials, and future treatment possibilities in AAV.

## Introduction

The systemic vasculitides are a heterogeneous group of diseases characterized by inflammation of blood vessels, resulting in tissue damage and end-organ dysfunction. Although relatively rare, if left untreated, these conditions lead to significant morbidity and mortality. While the etiology of the primary vasculitides is still unknown, a better understanding of their pathogenic mechanisms in combination with an expanded armamentarium of targeted therapeutics has led to significant advances in treatment. Among the primary systemic vasculitides, the treatment of anti-neutrophil cytoplasmic antibody (ANCA)-associated vasculitides (AAVs) has most notably benefited from these recent advances. Landmark studies in AAV showing the efficacy of B cell depletion have led to rapid clinical utilization of rituximab (RTX), an anti-CD20 monoclonal antibody which targets and depletes premature and mature B lymphocytes. However, once patients are in remission, there is controversy surrounding the appropriate timing, dose, frequency, duration, and long-term effects of this treatment. The purpose of this review is to provide an overview of the current information, recent advances, ongoing clinical trials, and future treatment possibilities in AAV.

## Induction of remission

The AAVs comprise granulomatosis with polyangiitis (GPA), microscopic polyangiitis (MPA), and eosinophilic granulomatosis with polyangiitis (EGPA). These multisystem, immune-mediated conditions primarily affect small blood vessels, most commonly small arteries, arterioles, capillaries, and venules of the lung and kidney. For patients with severe systemic AAV, the mainstay of remission induction treatment has included high-dose glucocorticoids and oral or intravenous (IV) pulse cyclophosphamide (CYC).

Although effective in 70–90% of patients with AAV ^1^, the use of CYC, particularly in refractory and relapsing disease, is limited owing to cumulative toxicity and serious adverse events which can include infertility, bladder hemorrhage, severe cytopenias, serious infection, and increased risk of malignancy. Because of these associated side effects, reducing the overall cumulative exposure to CYC during remission induction has been advocated. De Groot and colleagues demonstrated that pulse dose CYC induced remission of AAV as well as daily oral regimens while reducing the cumulative CYC dose by half and significantly decreasing the frequency of treatment-related leukopenia ^2^. Despite limited power to determine the effect of the evaluated induction regimens on relapse, longer-term follow-up identified that patients receiving pulse dose CYC had a twofold increased risk of relapse compared to those receiving daily oral CYC ^3^. This was particularly pronounced among those with anti-proteinase 3 antibodies (PR3-ANCA). As such, limiting CYC treatment may not be as beneficial for some patient subsets.

In AAV, CYC appears to exert a greater effect on B cells compared to T cells ^4^. B cells and their progeny appear to be central in the pathogenesis of AAV through the production of ANCA, as well as T cell co-stimulation and cytokine production ^5^. Furthermore, the number of circulating activated B cells correlates with disease activity ^6^. The identification of these pathogenic roles provided the framework for two landmark randomized trials to evaluate B-cell-targeted therapy for remission induction in patients with GPA and MPA.

The Rituximab vs. Cyclophosphamide in ANCA-associated Renal Vasculitis (RITUXVAS) trial included 44 patients with newly diagnosed GPA or MPA and renal involvement. In addition to receiving high-dose glucocorticoids, patients were randomized (3:1) to induction treatment with either RTX (375 mg/m ^2^) weekly for 4 weeks with two IV pulses of CYC or IV CYC pulses for 3–6 months followed by azathioprine (AZA) maintenance ^7^. Sustained remissions at 12 months were similar between the RTX (76%) and CYC/AZA (82%) groups, demonstrating the efficacy of RTX for remission induction in patients with AAV and significant renal dysfunction. Surprisingly, no difference in adverse events or mortality was observed. Extended trial follow-up data at 24 months ^8^ showed that the RTX-based induction without maintenance therapy had similar longer-term outcomes to the CYC/AZA regimen with regard to relapse (21% vs. 18%) and mortality (18% vs. 27%).

The Rituximab vs. Cyclophosphamide for ANCA-associated Vasculitis (RAVE) trial was a randomized, double-blind, double-dummy, placebo-controlled, non-inferiority trial evaluating a total of 197 patients with GPA or MPA ^9^. In contrast to RITUXVAS, RAVE included both newly diagnosed and relapsing patients comparing RTX 375 mg/m ^2^ weekly for 4 weeks to 2 mg/kg oral CYC for 3–6 months followed by AZA maintenance. All trial participants received high-dose glucocorticoids, which were tapered off according to a standardized protocol. The RAVE trial concluded that RTX was non-inferior to CYC/AZA for inducing remission at 6 months, with 64% of RTX patients and 53% of CYC/AZA patients reaching this endpoint. However, for GPA/MPA patients entering the study with relapsing disease, RTX appeared more efficacious with 67% (34/51) of patients reaching remission at 6 months compared to 42% (21/50) with CYC/AZA. Extended follow-up results showed similar long-term efficacy between the two regimens with 48% and 39% of patients in sustained remission at 12 and 18 months in the RTX arm and 39% and 33% in sustained remission at 12 and 18 months in the CYC/AZA arm, respectively ^10^.

Though it was anticipated that induction with RTX might be safer than CYC, it is of note that the number of adverse events in both the RITUXVAS and RAVE studies was similar between the two treatment arms ^7, 9^. Potential reasons may include identification of treatment-related complications due to concomitant high-dose glucocorticoids and short duration of follow-up preventing an observed difference in known long-term CYC-associated adverse events.

Taken together, the results of RITUXVAS and RAVE demonstrate RTX to be equivalent to CYC for remission induction of AAV among treatment-naïve patients and likely superior to CYC for relapsing disease.

## Remission maintenance

GPA and MPA, however, are chronic diseases, and up to 40–50% of patients can have a relapsing course despite immunosuppressive treatment. Traditionally, patients treated with a CYC induction regimen are transitioned to an oral conventional immunosuppressive agent, while glucocorticoids are gradually tapered. Trial data from the Cyclophosphamide vs. Azathioprine for Early Remission Phase of Vasculitis (CYCAZAREM) study found that early substitution with AZA following a minimum of 3 months of oral CYC therapy had similar overall outcomes to a 12-month oral CYC regimen ^1^. However, a slight numerical increase in relapse was observed in longer-term follow-up among those receiving shorter CYC courses ^11^. Further research is needed to determine if certain subsets of patients would benefit from a duration of CYC treatment tailored to their risk of relapse and likelihood of treatment-related morbidity ^11^. While AZA is the most commonly used immunosuppressive agent for remission maintenance, methotrexate has also shown similar efficacy ^12^, provided patients have adequate renal function. Mycophenolate mofetil, on the other hand, has been shown to be inferior to AZA for remission maintenance but remains an alternative for patients intolerant of other immunosuppressive options ^13^. In addition to conventional oral immunosuppressive medications, repeated administration of RTX may also be a viable option for remission maintenance.

The long-term follow-up data from the RITUXVAS and RAVE studies confirm that a single course of RTX is beneficial for induction. Unfortunately, relapse will occur in the majority of patients if they are not provided with maintenance treatment. This appears particularly true among those with upper respiratory tract disease, granulomatous infiltrates, or PR3-ANCA positivity ^14– 16^. Several open-label studies have evaluated the use of RTX to prevent relapse; unfortunately, heterogeneity in the timing, dose, and duration of treatment has prevented consensus on a preferred maintenance regimen. Smith *et al.* retrospectively studied relapsing GPA/MPA patients induced with RTX and then either observed until relapse or treated with fixed-interval RTX maintenance dosing of 1 g every 6 months ^17^. Over 2 years, 73% of the observed group had relapsed compared to only 12% receiving fixed-interval doses of RTX. Additional investigators have observed similar efficacy of pre-emptive dosing for reducing relapse rates and prolonging relapse-free survival ^16, 18– 21^, leading many clinicians to employ such treatment approaches.

Several prospective randomized trials are currently aimed at understanding the utility of RTX as maintenance therapy ( Table 1). MAINRITSAN (Maintenance of Remission using Rituximab in Systemic ANCA-associated vasculitis) is the first randomized trial to compare RTX and AZA in the maintenance of AAV ^22^. This open-label study randomized 115 patients in complete remission following standard induction with CYC and glucocorticoids to receive either RTX (500 mg on day 1 and 14, then at months 6, 12, and 18) or AZA (2 mg/kg/day for 12 months, 1.5 mg/kg/day for 6 months, then 1.0 mg/kg/day for last 4 months). At month 28, major relapse occurred in 29% (17/58) of patients receiving AZA maintenance compared to only 5% (3/57) with RTX. Although this study demonstrates RTX may be superior to AZA in maintaining remission at 2 years, it should be noted that the AZA dose was reduced starting (already) at 12 months, a schedule not frequently employed in clinical practice. Indeed, 41% of the relapses in the AZA group occurred after treatment cessation. Therefore, it is unknown if the difference in relapse rates would have been less striking if higher doses of AZA were maintained throughout the entire study period. In order to address this question, an international collaborative trial (RITAZAREM: Clinicaltrials.gov identifier NCT01697267) is ongoing. RITAZAREM will evaluate relapsing AAV patients randomized to either RTX 1 g every 4 months for five doses or AZA 2 mg/kg/day for 24 months, more closely paralleling typical clinical practice.

**Table 1.** Summary of ongoing clinical trials in ANCA-associated vasculitis.

Clinical trial	Identifier	Phase	Agent(s)	Status	Target Completion	Arms	Primary outcome
RITAZAREM	NCT01697267	3	RTX, AZA	Recruiting	12-2018	Exp: RTX 1g at 4,8,12,16 &20 months with standardized steroid taper Active Comp: AZA 2mg/kg/day with standardized steroid taper from month 4	Time from randomization to disease relapse (major or minor)
MAINRITSAN 2	NCT01731561	3	RTX	Active, not recruiting	2-2017	Exp: RTX 500mg D1 with steroid taper; ANCA and CD19+ count monitored q 3 months Re-dose if CD19 > 0/mm3 or ANCA increases or turns (+) Active Comp: RTX 500 mg D1, D15, M6, M12, M18 with steroid taper	Number of relapses (major and minor) at 28 months
MAINRITSAN 3	NCT02433522	3	RTX	Recruiting	1-2019	Exp: RTX 500 mg at randomization and q 6 months for 18 months Placebo Comp: placebo infusion q 6 months for 18 months	Relapse free survival at 28 months
PEXIVAS	NCT00987389	3	PLEX, GC	Recruiting	4-2018	Exp (1): PLEX, standard GC Exp (2): PLEX, reduced GC Active Comp (1): No PLEX, standard GC Active Comp (2): No PLEX, reduced GC	All-cause mortality, ESRD
BREVAS	NCT01663623	3	Belimumab	Active, not recruiting	1-2017	Exp: Belimumab 10mg/kg on D0, D14, D28, then every 28 days plus AZA 2mg/kg/day Placebo Comp: Placebo IV on D0, D14, D28 then every 28 days, plus AZA 2mg/kg/day	Time-to-first relapse
ABROGATE	NCT02108860	3	Abatacept	Recruiting	9-2018	Exp: Maintenance medication + Abatacept 125 mg subcutaneus weekly for 12 months + standardized GC taper Placebo Comp: Maintenance medication + sterile subcutaneous injection weekly for 12 months + standardized GC taper	Treatment failure after 12 months of study treatment (relapse, disease worsening, failure to achieve BVAS/ WG ≤1)
CLEAR	NCT01363388	2	CCX168	Completed		Exp (1): Standard induction [CYC or RTX] + CCX168 30 mg twice daily + low-dose initial prednisone (20 mg) Exp (2): Standard induction [CYC or RTX] + CCX168 30 mg twice daily + no initial prednisone Placebo Comp: Standard induction [CYC or RTX] + placebo + high-dose initial prednisone (60 mg)	Safety (adverse events) and efficacy (assessed by BVAS/WG)
CLASSIC	NCT02222155	2	CCX168	Active, not recruiting	9-2016	Exp (1): CCX168 10 mg BID + standard induction Exp (2): CCX168 30 mg BID + standard induction Placebo Comp: Placebo BID + standard induction	Change in BVAS/WG from randomization to 12 weeks

AZA, azathioprine; BID, twice daily; BVAS/WG, Birmingham Vasculitis Activity Score for Wegener’s Granulomatosis; Comp, comparator arm; CYC cyclophosphamide; D, day; ESRD, end-stage renal disease; Exp, experimental arm; GC, glucocorticoids; M, month; PLEX, plasma exchange; RTX, rituximab;

Although considered to be a potentially safer alternative to CYC, the delayed adverse effects of long-term RTX maintenance in AAV are unknown. While some studies show that repeat dosing is well tolerated ^19, 21^, others demonstrate notably increased adverse events, including serious infection, late-onset neutropenia, and hypogammaglobulinemia ^20, 23^. A tailored approach to guide pre-emptive treatment decisions based on serial B lymphocyte and ANCA titer monitoring has been suggested by Cartin-Ceba and colleagues ^19^. However, other investigators have demonstrated that these parameters are not uniformly consistent in relapse prediction ^17, 18, 24^. MAINRITSAN 2 (ClinicalTrials.gov identifier NCT01731561) was organized to assess this critical knowledge gap and has been designed to compare two treatment regimens for RTX maintenance; 500 mg every 6 months vs. 500 mg whenever CD19 ^+^ lymphocytes re-populate or when ANCA titers become positive or rise. Extended follow-up of this trial with an additional 18 months of fixed dosing RTX at 6-month intervals vs. placebo is also planned (MAINRITSAN 3: ClinicalTrials.gov identifier NCT02433522). These two trials will greatly advance the understanding of RTX use in remission maintenance, and results are expected in the next 3–5 years.

## Adjunct plasma exchange

The overall benefit of plasma exchange (PLEX) in severe AAV remains uncertain. Initial evidence of efficacy with PLEX was seen in a small randomized controlled trial by Pusey and colleagues evaluating patients with anti-GBM-negative renal vasculitis ^25^. In this trial, dialysis-independent patients improved with either standard drug therapy with adjunctive PLEX or drug therapy alone, regardless of initial serum creatinine. However, patients who were dialysis dependent at study entry experienced greater likelihood of renal recovery with the addition of PLEX compared to standard drug therapy. In a larger trial, Jayne *et al.* ^26^ evaluated the effect of adjunctive therapy with PLEX or IV methylprednisolone (MP) in 137 patients with severe renal vasculitis (serum creatinine >5.8 mg/dL or dialysis dependent) receiving standard treatment with oral CYC and oral prednisone. Those randomized to PLEX had a higher rate of renal recovery and independence from dialysis compared to those treated with IV MP at both 3 months (69% vs. 49%, respectively) and 12 months (43% vs. 19%, respectively).

Although short-term outcomes were encouraging, long-term benefits are unclear, as subsequent follow-up of this cohort for a median of 3.95 years did not identify a significant difference between PLEX and IV MP among the outcomes of end-stage renal disease (ESRD) or mortality ^27^. A meta-analysis comprising 387 patients with AAV from nine randomized trials found that patients treated with PLEX had a significant reduction in dialysis dependence (relative risk [RR] 0.64; 95% confidence interval [CI]: 0.47–0.88) but not mortality (RR 1.01; 95% CI: 0.71–1.43) ^28^. However, the authors note that the cumulative evidence was inadequate to conclude that PLEX effectively decreases ESRD because more than 1478 patients would have been needed to determine a 25% RR reduction with appropriate confidence ^28^.

In addition to its use for severe renal vasculitis, PLEX has been considered for the treatment of AAV with pulmonary complications from diffuse alveolar hemorrhage (DAH). The use of PLEX in DAH, however, is limited to uncontrolled observational studies and results are conflicting. Klemmer and colleagues ^29^ found that prompt initiation of PLEX in addition to standard induction therapy for AAV led to resolution of DAH in all 20 patients retrospectively reviewed. Resolution occurred after an average of six treatments, and no treatment-associated complications were seen. Nevertheless, the absence of a control group prevents concluding whether PLEX itself led to such improvements. In a recent retrospective study, Cartin-Ceba *et al*. evaluated the outcomes of 73 patients with AAV and DAH and did not observe a benefit in achieving complete remission at 6 months between adjunctive PLEX and standard induction therapy (odds ratio [OR] 0.49; 95% CI: 0.12–1.95) ^30^. The authors further evaluated 11 uncontrolled studies, including their own, and found resolution of DAH and survival to hospital discharge was achieved in 66% (69/104) of patients treated with PLEX compared to 75% (51/68) without, tempering the likelihood that PLEX is necessary in this population ^30^.

To address these uncertain roles of PLEX, a randomized controlled trial recruiting patients with severe renal vasculitis and/or diffuse alveolar hemorrhage secondary to AAV has been developed and is underway (PEXIVAS, ClinicalTrials.gov identifier NCT00987389). Currently, the precise role of PLEX as adjunctive therapy for remission induction in AAV remains unclear. However, given the severity of disease, it is still considered by some experts to be a reasonable option for patients with severe renal disease or dialysis dependence at the time of diagnosis. The results of PEXIVAS are highly awaited to provide further insight and recommendations.

## Future therapeutic investigations

Until this past decade, CYC was considered the only reliable induction agent for AAV. The success of non-selective B cell depletion with RTX in AAV has opened the door for the next generation of targeted therapies focusing on the innate and adaptive immune system ( Table 1).

B-cell-activating factor (BAFF), also known as B lymphocyte stimulator (BLyS), appears to have a key role in the stimulation of B cell proliferation and the promotion of immature B cell survival. Increased BAFF levels favor the selection of autoreactive B cells, leading to autoantibody production ^31^, and have been associated with autoimmune conditions such as systemic lupus erythematosus ^32^. Elevated BAFF levels have also been seen in patients with GPA, most notably among active untreated patients ^33^, and ANCA-stimulated neutrophils promote B cell survival through the release of BAFF ^34^. Interestingly, researchers have observed BAFF levels increasing after B cell depletion with RTX in AAV models ^34^. This finding demonstrates that BAFF may be an integral factor in the survival of autoreactive B cells and likely facilitates disease chronicity and relapse.

Belimumab is a monoclonal antibody directed against BAFF that has been approved for the treatment of systemic lupus erythematosus. This targeted BAFF inhibitor is currently being investigated in a phase III multicenter randomized trial evaluating the efficacy and safety of this medication in combination with AZA for the maintenance of remission in GPA and MPA (BREVAS: ClinicalTrials.gov identifier NCT01663623). Additional B cell survival factors are also under investigation in AAV. Blisibimod, a fusion protein that binds both soluble and membrane-bound BAFF, is currently being considered for a phase II trial for remission induction in non-severe AAV patients receiving concomitant methotrexate.

Although B cell dysregulation has gained center stage in the treatment of AAV, abnormal circulating and lesional T cell activation may also play a role in pathogenesis ^35^. An open-label study evaluating abatacept, a fusion protein that blocks the co-stimulatory signal needed for T cell activation, showed disease improvement in 90% (18/20) and steroid discontinuation in 73% (11/15) of patients with non-severe AAV. However, 30% (6/20) had to terminate the study owing to increases in disease activity ^36^, portending the likelihood that T cell activation alone is unlikely to be the critical pathway for disease control. A multicenter, phase III, double-blind, placebo-controlled trial further evaluating the use of abatacept in relapsing non-severe AAV is ongoing (ABROGATE: ClinicalTrials.gov identifier NCT02108860).

Gusperimus, a synthetic immunosuppressive drug derived from the antitumor antibiotic spergualin, also modulates lymphocyte function in addition to other poorly understood pathways and is a candidate drug for use in AAV ^37^. Two open-label studies have noted disease improvement among 70–95% of patients with refractory GPA ^38, 39^. Follow-up data have also demonstrated continued efficacy and minimal toxicity with up to 5 years of treatment ^40, 41^. A clinical trial comparing gusperimus to conventional therapy is currently being designed.

Complement activation, particularly component C5a, has also been recently implicated in the pathogenesis of AAV. C5a is a potent inflammatory mediator as well as a strong chemoattractant and neutrophil activator ^42^. C5a acts as a priming agent for neutrophils, resulting in increased surface expression of the auto-antigens proteinase 3 (PR3) and myeloperoxidase (MPO). These auto-antigens interact with ANCA and subsequently stimulate the release of factors that activate the alternative complement pathway, further perpetuating cleavage of C5 and increases in C5a levels, leading to an amplification loop of ANCA-mediated neutrophil activation ^42, 43^. In animal models, both C5a knockout and blockade of the neutrophil C5a receptor have been shown to be protective against ANCA-induced glomerulonephritis ^44^.

An orally administered inhibitor of C5a receptor (CCX168) has recently completed phase II investigation in Europe (CLEAR: ClinicalTrials.gov identifier NCT01363388). The CLEAR study evaluated three arms ( Table 1). All patients received standard induction with either CYC or RTX and were randomized to one of the following: CCX168 + low-dose (20 mg) initial prednisone, CCX168 + no initial prednisone, or placebo + high-dose (60 mg) initial prednisone. Preliminary data show that both treatment groups receiving CCX168 were non-inferior to the standard induction and high-dose prednisone. This was demonstrated by complete remission obtained at 12 weeks in 75% AAV patients in the high-dose prednisone + placebo group, 86% in the CCX168 + low-dose prednisone (p=0.005 for non-inferiority), and 81% in the CCX168 + no prednisone group (p=0.02 for non-inferiority). While this is clearly an important milestone for complement-targeted therapies, complete data analysis is yet forthcoming and the North American phase II trial of low- vs. high-dose CCX168 in addition to standard induction in AAV is still ongoing (CLASSIC: ClinicalTrials.gov identifier NCT02222155).

Finally, evidence is accumulating that inflammatory cytokines may play a role in the pathogenesis and activity of AAV. Interleukin (IL)-6 levels are increased in both serum and histopathologic samples from patients with active AAV ^45^. Furthermore, higher serum IL-6 levels appear to be associated with patients with frequent relapse and more severe organ damage ^45^. IL-6 blockade with tocilizumab has shown promise in limited case reports ^45, 46^ and requires further evaluation. Similar to IL-6, increased serum levels of IL-17 and IL-23 have been observed in patients with AAV compared to healthy controls ^47^. Additionally, AAV patients with higher IL-23 levels had more active disease and higher ANCA titers ^47^. Both cytokines are associated with T helper 17 (Th17) cells, a T cell subset critical in mediating autoimmune disease. Specifically, IL-23 enhances the differentiation of T cells towards the Th17 subset and further assists in maintaining the production of IL-17, which itself is a proinflammatory cytokine with pleiotropic action. Ustekinumab (anti-IL-12/23) and secukinumab (anti-IL-17A) are both currently commercially available for the treatment of plaque psoriasis. These targeted anti-inflammatory cytokine therapies have yet to be trialed in AAV, but plans for future research are being considered.

## Conclusions

The success of RTX in AAV is unequivocal, and its rapid utilization in these conditions highlights the four-decade-long unmet need for reliable alternatives to CYC. Induction with RTX can be considered a first-line option for both treatment-naïve and relapsing patients, particularly in young patients wanting to preserve fertility and for patients with a history, or at increased risk, of malignancy. The benefit of PLEX in induction management is still unclear but is considered reasonable in patients presenting with severe renal dysfunction. Although prospective trial evidence is limited, it is clear that maintenance treatment is needed following RTX induction in the majority of patients. In the next 5 years, the results from ongoing clinical trials will provide guidance and clarity to several clinical questions that have arisen in the post-RTX era.

Paralleling the advances made in rheumatoid arthritis treatment over the past two decades, the treatment of vasculitis in general, and AAV in particular, has now entered the increasingly expansive arena of targeted therapeutics. While phenotypically similar, the immune pathway targets at the individual level in immune-mediated diseases are extraordinarily complex and likely the reason for the variability in clinical outcome among large therapeutic trials. Significant advances have been made in understanding AAV at a population level, and this will ultimately lead to better understanding at the individual level to allow tailoring of treatments and a more personalized approach to management. In addition to generating novel therapeutics to newly identified pathway targets, the next decade will also likely bring combined therapeutic approaches where biologic agents are trialed in combination or succession.
